# High-Dose Convalescent Plasma for Treatment of Severe COVID-19

**DOI:** 10.3201/eid2805.220191

**Published:** 2022-05

**Authors:** Daniele Focosi, Arturo Casadevall

**Affiliations:** Pisa University Hospital, Pisa, Italy (D. Focosi);; Johns Hopkins School of Public Health and School of Medicine, Baltimore, Maryland, USA (A. Casadevall)

**Keywords:** COVID-19, 2019 novel coronavirus disease, coronavirus disease, severe acute respiratory syndrome coronavirus 2, SARS-CoV-2, viruses, respiratory infections, zoonoses

**To the Editor:** We commend our colleagues in Brazil for completing a multicenter, open-label, randomized controlled trial (RCT) of coronavirus disease (COVID-19) convalescent plasma (CCP) against wild-type severe acute respiratory syndrome coronavirus 2 (SARS-CoV-2) (*1*). This RCT had some strengths, including use of high-dose CCP (600 mL CCP for 3 days at a median neutralizing antibody titer of 1:128). The overall results were negative, but the authors caution that this finding probably reflects inclusion of patients late in disease, as evident by enrollment criteria (oxygen saturation <93%, partial pressure of oxygen/fraction of inspired oxygen <300, or need for mechanical ventilation), median transfusion at day 9 after symptom onset, 100% seropositivity, and 35% requiring hemodialysis at enrollment. The severity of disease in those patients means that disease was driven by inflammation as opposed to ongoing virus replication. To date, 2 CCP RCTs have shown benefit, 1 that provided outpatient treatment (D. Sullivan, et al., unpub. data, https://www.medrxiv.org/content/10.1101/2021.12.10.2126748v1) and 1 that provided inpatient treatment within 3 days of symptom onset (*2*). Hence, we caution against negative conclusions about the efficacy of CCP based on these data.

We find it remarkable that despite late CCP use, the authors observed a lower mortality rate among CCP-treated patients (31%) than controls (35%), given that the prevailing view is that this therapy functions as an antiviral and should not be effective in late disease. A similar finding is apparent in most other RCTs of hospitalized patients (*3*). This reduced mortality rate did not reach statistical significance because of the low sample size, which was estimated by assuming a 50% reduction in mortality rate from the intervention, much higher than that assumed in the RCTs of anti-spike monoclonal antibodies (typically in the range of 20%); further, recruitment was halted at 110 out of 120 patients. A recent article suggests that there is a population with high World Health Organization severity scores that benefits from CCP (*4*). We wonder if the authors can reanalyze their data by using the treatment benefit calculator (https://covid-convalescentplasma-tbi-calc.org) (*4*) to gain more insight into whether a small subset of patients benefited from CCP.
